# Dipteran Carboxymethyl Chitosan as an Inexhaustible Derivative with a Potential Antiproliferative Activity in Hepatocellular Carcinoma Cells

**DOI:** 10.1155/2020/4396305

**Published:** 2020-09-28

**Authors:** Rana M. Abdel Rahman, Hedayat Abdel Ghaffar, Afrah F. Alkhuriji, Mahmoud I. Khalil, Noha Amaly, Attalla F. El-Kott, Ahmed S. Sultan

**Affiliations:** ^1^Zoology Department, Faculty of Science, Damanhour University, Damanhour, Egypt; ^2^Zoology Department, Faculty of Science, Alexandria University, Alexandria, Egypt; ^3^Department of Zoology, College of Science, King Saud University, Riyadh, Saudi Arabia; ^4^Department of Biological Sciences, Faculty of Science, Beirut Arab University, Lebanon; ^5^Molecular Biology Unit, Zoology Department, Faculty of Science, Alexandria University, Alexandria, Egypt; ^6^Polymeric Materials Research Department, Advanced Technology and New Materials Research Institute (ATNMRI), City of Scientific Research and Technological Applications (SRTA-City), Alexandria, Egypt; ^7^Biology Department, Faculty of Science, King Khalid University, Abha, Saudi Arabia; ^8^Biochemistry Department, Faculty of Science, Alexandria University, Alexandria, Egypt

## Abstract

Traditional folk therapies indicate that insects have diverse medicinal potentials. However, the therapeutic application of insect chitosan and its derivatives has not been explored. To investigate the application of chitosan and its derivatives, the carboxymethyl derivative of chitosan (CM-Ch) was extracted from two dipteran larvae species, *Chrysomya albiceps* and *Sarcophaga aegyptiaca*. The degree of deacetylation (DD) and CM-Ch functional groups were validated using Fourier-transform infrared (FTIR) spectroscopy analysis and proton nuclear magnetic resonance spectroscopy (^1^H NMR), respectively. The molecular weight was estimated using MALDI-TOF MS analysis. The effect of CM-Ch on the morphology and proliferation of human liver HepG2 cancer cells was assessed. IC_50_ of CM-Ch induced significant growth-inhibitory effects in HepG2 cells. CM-Ch treatment altered the morphology of HepG2 in a dose-dependent manner and induced apoptosis in a caspase-dependent manner. CM-Ch treatment showed no signs of toxicity, and no alterations in liver and kidney biochemical markers were observed in albino rats. A CM-Ch derivative from commercial crustacean chitosan was used to assess the efficacy of the insect-derived CM-Ch. The data presented here introduce insect CM-Ch as a promising, inexhaustible, safe derivative of chitosan with antitumor potential in liver cancer. This is the first report highlighting the anticancer activity of insect CM-Ch in hepatocellular carcinoma cells.

## 1. Introduction

Primary liver cancer is a major public health problem that causes around half a million deaths per year worldwide. Moreover, the incidence of primary liver cancer is increasing. Hepatocellular carcinoma (HCC) is the most common primary liver cancer malignancy [1] and is one of the most predominant cancer types in Egypt [2]. Natural products and their derivatives have been recognized as essential therapeutic sources [3]. Historical and traditional folk therapies have revealed that insects present diverse biological activities and medicinal potentials. Investigating relatively unexplored habitats and novel organisms can maximize the success of drug discovery efforts. Indeed, very few insects have been screened for therapeutically relevant molecules [4].

Various substances extracted from insects, including chitin, antibacterial peptides, and polysaccharides, have been studied [5, 6]. Chitin is widely distributed in the cuticles of crustaceans and insects and in the cell walls of some fungi and microorganisms [7]. Chitosan is the alkaline deacetylated derivative form of chitin [8]. The distinctive feature of commercially available crustacean raw chitin is it contains a large amount of other substances, including fatty acids, lipids, calcium phosphate, and carbonates. The commercial extraction of chitin from traditional resources constitutes a high natural environmental load that is restricted by season [9, 10]. Owing to the enormous biodiversity of insects, more attention has been focused on the commercial preparation of chitin and chitosan from insects for applications on a wide biomedical scale [6, 11]. Chitosan possesses biological characteristics including biocompatibility, nonimmunogenicity, antimicrobial activity, and biodegradability. Accordingly, chitosan has been used in food, biotechnology, and pharmaceutical industries as an antifungal, antibacterial, antitumor, immune-enhancing, anticholesteremic, and antithrombogenic agent and for the amelioration of diabetes and painful diabetic neuropathy [12–15]. Additionally, combining chitosan with other compounds is an efficient and safe adjuvant for the hepatitis B vaccine in a mouse model [16]. Nevertheless, chitosan application is severely limited because it is insoluble in neutral or alkaline pH because of its stable crystalline structure, low absorbability, high molecular weight, and high viscosity [17]. Recently, carboxymethyl chitosan (CM-Ch) has received attention because of its solubility in water, biocompatibility, antibacterial and antitumor properties, and low toxicity [8, 18].

*Chrysomya albiceps* and *Sarcophaga aegyptiaca* are prevalent necrophagous species of medical importance worldwide. These species exhibit a broad worldwide distribution and are characterized by having a short life cycle, which is a major requirement for controlled laboratory rearing [19, 20].

This study was designed to investigate the potential of CM-Ch, as an insect extract, to be used as a biomedical approach for liver cancer therapy. To this end, we developed a cheap and commercially feasible chitosan extraction procedure from two unexplored dipterous insect sources, *C. albiceps* and *S. aegyptiaca*. We then systematically evaluated the potential anticancer activities of insect CM-Ch on the HepG2 human hepatocellular carcinoma cell line and performed a preliminarily investigation of the acute toxicity of CM-Ch *in vivo.*

## 2. Materials and Methods

### 2.1. Insect Collecting and Rearing

Prepupal stage *C. albiceps* and *S. aegyptiaca* were gathered from exposed rabbit carcasses at the botanical garden of the Faculty of Science, Moharrem Bey District, Alexandria, Egypt. The collected prepupae were transferred into a bowl containing dry autoclaved sawdust for pupation. Pupae were transferred to adult cages. Adult flies were maintained in rearing cages at room temperature. Each cage consisted of water, granular sucrose, powdered yeast, and powdered milk. Each cage was supplemented with a dish of 100 g of fresh beef liver that was removed once sufficient eggs were laid, in the case of *C. albiceps*, or sufficient larvae emerged, in the case of viviparous *S. aegyptiaca*. Newly hatched first instar larvae were transferred into 100 mL jars containing 50 g of cow liver. Each jar was placed in a larger beaker containing a 2 cm bed of dry sawdust and covered with a fine nylon mesh. Jars were kept at 25 ± 2°C, in 60% ± 10% humidity, and a photoperiod of 12 h : 12 h. Third instar larvae were stored at −20°C for chitosan extraction.

### 2.2. Chitosan Extraction

Chitosan was extracted from *S. aegyptiaca* and *C. albiceps* larvae as described previously [21] with some modifications. In brief, larvae were washed with 15% aqueous NaCl and dried at 50°C. To retrieve crude chitin from the cuticle, larvae were treated with 1 mol/L aqueous NaOH for 6 h at 100°C. The retrieved crude chitin was rinsed with water until reaching pH 7.4, filtered with a mesh, and freeze-dried. To obtain crude chitosan, crude chitin was N-deacetylated with 50% w/v NaOH solution for 6 hours at 125°C. The mixture was rinsed several times with water to reach required pH and filtered using a mesh. Chitosan obtained was stored at −20°C.

### 2.3. Preparation of Carboxymethyl Chitosan (CM-Ch)

CM-Ch was extracted from *Sarcophaga* and *Chrysomya* larvae and commercial chitosan (Sigma-Aldrich) as previously described [22]. In brief, 10 g of chitosan from *Sarcophaga*, *Chrysomya*, and commercial chitosan were dispersed in 50 mL distilled water for 30 min, then 50 mL of isopropyl alcohol was added, and the samples spun at 300 rpm at room temperature for 30 min. Aliquots of 10 M NaOH (15 mL) were added prior to spinning for 45 min. Monochloroacetic acid (30 g) was added, and the mixture was incubated at 55°C for 1 h. The solution was filtered and washed with 80% EtOH (v/v) several times to remove chloroacetic acid. The white-yellow powder obtained was dried for 24 h at 50°C.

### 2.4. Characterization of Chitosan and CM-Ch

#### 2.4.1. Fourier-Transform Infrared (FTIR) Spectroscopy Analysis

Infrared spectroscopy measurements were performed in the transmission mode using an FTIR spectrometer (Shimadzu FTIR-8400S, Japan). FTIR spectrum (frequency range of *λ* = 400–4000 cm^−1^) was determined for pellets containing 2 mg of chitosan and CM-Ch samples in 200 mg of KBr.

#### 2.4.2. Determination of the Degree of Deacetylation (DD) in Chitosan

DD was assessed using FTIR spectroscopy as described previously [23]. The DD was calculated using Baxter's equation [24]:

(1) $$\begin{array}{c} \text{DD}=100-\;\left[\frac{A_{1655}\;}{A_{3450}}\times \frac{100}{1.33}\right], \end{array}$$

where *A*_1655_ is the absorbance of the amide-I bond as a measure of the N-acetyl group content and *A*_3450_ of the N-H bond. The factor “1.33” denotes the value of the *A*_1655_/*A*_3450_ ratio for fully N-acetylated chitosan.

#### 2.4.3. Determination of the Molecular Weight of Chitosan

The intrinsic viscosity [*η*] of 0.1% chitosan in 1% v/v AcOH was measured using a viscometer. The viscosity average-molecular weight (M_wt_) was calculated using the Mark–Houwink relationship [25]: [*η*] =  *K*.*M*_wt_^*a*^, where “*η*” is the intrinsic viscosity and “*K*” and “*a*” are calculated as

(2) $$\begin{array}{c} K=1.64\times 10^{-30}\times {\left(\%\text{DD}\right)}^{14},\\ a=-1.02\mathrm{\times 1}0^{\mathrm{-2}}\times \left(\%\text{DD}\right)+1.82. \end{array}$$

#### 2.4.4. Proton Nuclear Magnetic Resonance (^1^H NMR) Spectroscopy

Water-soluble CM-Ch samples were dissolved in 1% DMSO. ^1^H NMR spectrum of CM-Ch samples was recorded using a JEOL JNM-ECA-500 spectrometer under a static magnetic field of 500 MHz at 25°C.

#### 2.4.5. Determination of CM-CH Molecular Weight by MALDI-TOF MS Analysis

The molecular weights of CM-Ch samples were determined by MALDI-TOF MS using 2,5-dihydroxybenzoic acid (DHB) as a sample matrix. The experiment was performed in an ultraflex TOF/TOF (Bruker Daltonics, Germany) in the positive reflection mode (400–6000 m/*z*).

### 2.5. Cell Culture

HepG2 liver cancer cells were obtained from ATCC (USA) and maintained in the DMEM medium (Gibco) supplemented with 10% fetal bovine serum, 100 U/mL penicillin, and 100 *μ*g/mL streptomycin at 37°C in a 5% CO_2_ incubator.

### 2.6. MTT Assay

Cells were seeded (2 × 10^5^ cells/per well) in a 96-well plate in complete DMEM medium. Cells were treated with increasing concentrations (0–1200 *μ*g/mL) of different CM-Ch. After 48 h, fresh medium, supplemented with 10 *µ*L of 12 mM MTT (3-(4,5-dimethylthiazol-2-yl)-2,5-diphenyltetrazolium bromide), was added, and cells were incubated for 4 h at 37°C. The reaction was stopped after the addition of 100 *µ*L SDS-HCl. The microplate was incubated for 18 h at 37°C. Absorbance (*A*) was read at 545 nm. The viability percentage was calculated using the formula ((*A*_Treated_ − *A*_Blank_)/(*A*_Control_ − *A*_Blank_) × 100). IC_50_ was determined using a sigmoidal curve.

### 2.7. Morphological Analysis

Cells were subjected to increasing concentrations (1–1000 *µ*g/mL) of CM-Ch for 24–72 h. Images were captured using an inverted phase contrast microscope at 400×. Digital images were acquired with a Kodak microscopic digital camera.

### 2.8. Enzyme-Linked Immunosorbent Apoptosis Assay (ELISA)

Cells were seeded (2 × 10^4^ cells^/^per well) in a 96-well plate in complete DMEM medium. After 24 hrs, cells were treated with different CM-Ch and incubated for an additional 48 h. A cell death detection ELISA plus kit (Roche, USA) was used to quantify histone release from fragmented DNA. In brief, cells were lysed at room temperature for 30 min. The lysates were centrifuged, and supernatant samples were incubated with antihistone biotin and anti-DNA peroxidase at room temperature for 2 h. After washing, a substrate solution (2,2′-azino-di(3-ethylbenzthiazolin-sulphuric acid) was added, and samples were incubated for 20 min at room temperature. Absorbance (OD_405_) was read using an ELISA reader (SpectraMax Plus).

### 2.9. Caspase-3 Activity

Caspase-3 activity was assayed calorimetrically following the manufacturer's protocol using the caspase-3 assay kit (BioVision, Inc., CA, USA). Cells (5 × 10^6^ cells/well) were treated with or without CM-Ch and lysed with HEPES lysis buffer (10 mM HEPES pH 7.4, 2 mM EDTA, 0.1% 5 mM CHAPS, 350 *μ*g/l PMSF, and 5 mM DTT). Lysates were centrifuged to remove cellular debris. Samples were supplemented with caspase-3 substrate (Ac-DEVD-AFC) for 1 h at room temperature. The reaction was stopped with 1N HCl. The absorbance (OD_405_) was read using a spectrophotometer (Jenway Spectrophotometer, UK).

### 2.10. LD_50_ Determination

Lethal doses can be predicted from IC_50_ values [26]. A good estimate of the starting doses for *in vivo* acute oral toxicity tests can be obtained using the following equation:

(3) $$\begin{array}{c} \text{log}\left(LD_{50}\right)=0.435\times \text{log}\left(IC_{50}\right)+0.625. \end{array}$$

### 2.11. CM-Ch Oral Toxicity Study

#### 2.11.1. Animal Study

Healthy male albino rats (8 weeks, ∼90 g) were used for the toxicity study. Animals were obtained from the Animal House of Alexandria University, Egypt. They were fed ad libitum with a standard diet and had free access to water. The animals were kept in standard polypropylene cages with a stainless-steel top grill and maintained at 23 ± 2°C under conditions of 5 ± 10% humidity and a 12 h light/dark cycle. Animals were adapted for three weeks prior to the commencement of the experiment. The use of laboratory animals and experimental procedures were conducted in accordance with the guidelines of the Animal Ethics Committee of Alexandria University, Egypt.

#### 2.11.2. Dosing

Twenty rats were divided into four groups of five rats each. Three groups were given a single oral dose of half LD_50_ of different CM-Ch preparations at the start of the experimental period (15 days) [27]. One group served as the control and received a single oral dose of saline. Rats were observed daily for any clinical signs and abnormal physical or behavioral changes. Mortality was monitored during the experimental period. Blood samples were collected on day 15 and used for further analysis.

#### 2.11.3. Body and Organ Weights

The body weights of rats were measured at least twice weekly. At the end of the experimental period, rats were anesthetized using cotton wool soaked in ether, and their final body weights were recorded. The liver, kidney, and spleen of rats were excised immediately. The organs were immediately weighed, and the organ weight ratio was calculated using the following equation:

(4) $$\begin{array}{c} \text{organ weight ratio}=\frac{\text{organ weight }\left(\text{g}\right)}{\text{body weight }\left(\text{g}\right)}\;\times \;100\text{.} \end{array}$$

#### 2.11.4. Determination of Biochemical Parameters

Blood samples were collected from the portal vein and kept to clot at room temperature for 30 min prior to centrifugation at 3000 rpm for 10 min to separate serum from clotted blood. The serum was collected for biochemical tests. Separated sera were used to evaluate kidney and liver functions. For the kidney, serum urea and serum creatinine were assessed as previously described [28, 29]. For the liver, serum aspartate aminotransferase (AST) and serum alanine aminotransferase (ALT) were assessed using a commercially available kit and following the instruction manual (BioSystems, Spain).

### 2.12. Statistical Analysis

Data are presented as means ± standard error of the mean (SEM) from at least three independent experiments. Data were subjected to one-way analysis of variance (ANOVA) using SPSS 10.0 software (SPSS, Chicago, IL, USA). The *p* value was calculated vs. control cells: ^*∗*^*p* < 0.05, ^∗∗^*p* < 0.01, ^∗∗∗^*p* < 0.005, and ^∗∗∗∗^*p* < 0.001.

## 3. Results

### 3.1. Characterization of Chitosan and Carboxymethyl Chitosan (CM-Ch)

**Figure 1:**
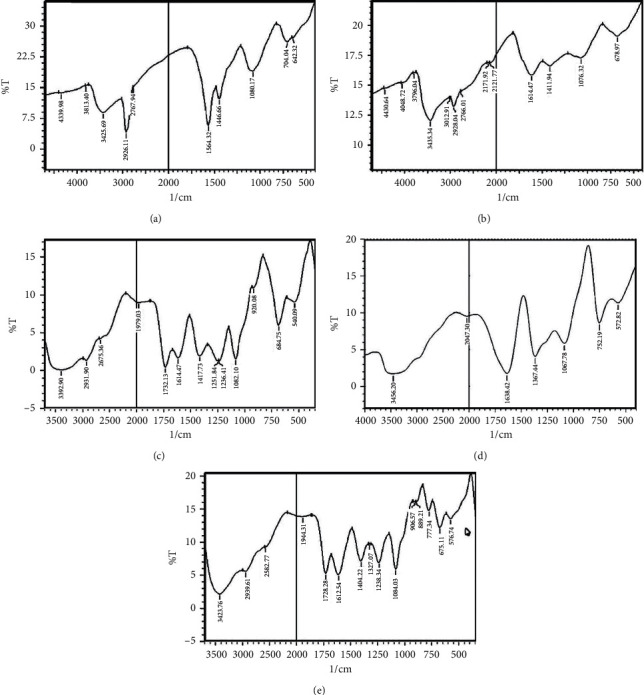
FTIR spectra of chitosan and carboxymethyl chitosan (CM-Ch). Spectra of chitosan extracted from *Chrysomya albiceps* (a) and *Sarcophaga aegyptiaca* (b). Spectra of the CM-Ch prepared from *C. albiceps* (c), *S. aegyptiaca* (d), and commercial crustacean chitosan (e).

The FTIR spectra of chitosan extracted from *C. albiceps* and *S. aegyptiaca* are shown in Figures 1(a) and 1(b), respectively. FTIR analysis results of the CM-Ch preparations from chitosan extracted from both flies and commercial chitosan are shown in Figures 1(c)–1(e). The FTIR vibration bands of chitosan extracted from *C. albiceps* and *S. aegyptiaca* and FTIR vibration bands of CM-Ch prepared from chitosan extracted from *C. albiceps* (Ca-CM-Ch), *S. aegyptiaca* (Sa-CM-Ch), and commercial chitosan (sy-CM-Ch) are shown in Table 1.

From the absorbance ratio *A*_1655_/*A*_3450_, the degree of deacetylation (DD) of chitosan extracted from *C. albiceps* and *S. aegyptiaca* was 84% and 90%, respectively. The viscosity average-molecular weights of chitosan extracted from *C. albiceps* and *S. aegyptiaca* larvae were 2.44 and 2.6 KDa, respectively. Peaks at 1614 and 1594 cm^−1^ were observed (Figures 1(a) and 1(b)), which were attributed to axial stretching of the C=O bond of the acetamide (-CONH-) group of the acetylated chitosan units. The C-O stretching vibration was recorded at 1080 and 1076 cm^−1^. The peaks at 3425/3435 and 1446/1411 cm^−1^ are characteristics for NH_2_ and C-N of the amine group, respectively. The peak intensity, characteristic for amine, at 3425 cm^−1^ for *C. albiceps* chitosan showed a lower intensity than that of *S. aegyptiaca* (3435 cm^−1^), which is consistent with the DD% results. FTIR spectra of the CM-Ch (Figures 1(c) and 1(d)) revealed new peaks at 1728 cm^−1^, which is not in the spectra of chitosan extracted from both flies (Figures 1(a) and 1(b)). The spectrum of the CM-Ch prepared from commercial chitosan did not reveal a peak at 1728 cm^−1^ (Figure 1(e)). Additionally, a broadened and shifted peak at 3425 cm^−1^, due to the grafted carboxylate group, is evidence of successful CM-Ch formation (Figures 1(c)–1(e)).

**Figure 2:**
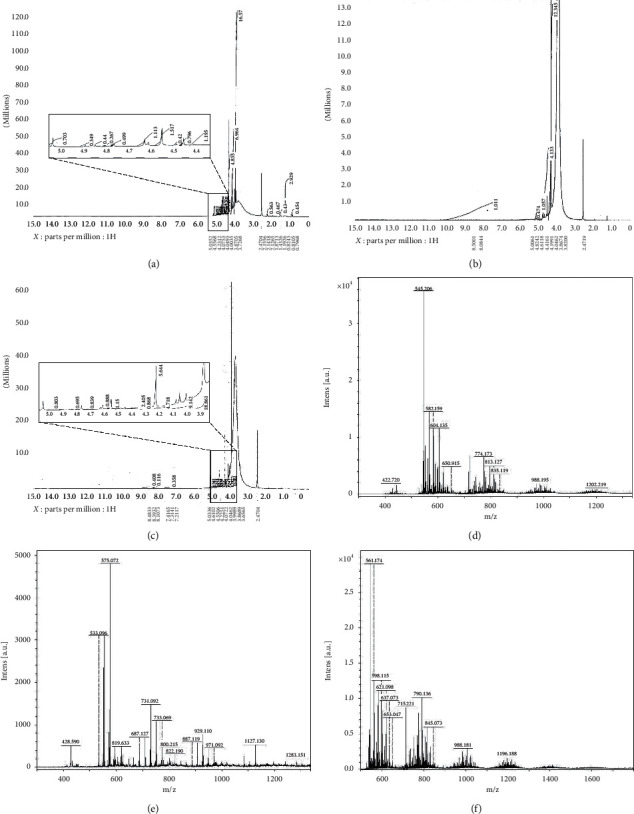
^1^H NMR spectra and molecular weights of carboxymethyl chitosan (CM-Ch). H^1^NMR spectra of *Chrysomya albiceps* CM-Ch (Ca-CM-Ch) (a), *Sarcophaga aegyptiaca* CM-Ch (Sa-CM-Ch) (b), and commercial chitosan CM-Ch (sy-CM-Ch) (c). Molecular weights of Ca-CM-Ch (d), Sa-CM-Ch (e), and sy-CM-Ch (f).

^1^H NMR analysis was used to further confirm CM-Ch preparations. The ^1^H NMR spectrum of CM-Ch prepared from *C. albiceps* (Ca-CM-Ch), *S. aegyptiaca* (Sa-CM-Ch), and commercial chitosan (sy-CM-Ch) is shown in Figures 2(a)–2(c). The chemical shifts at 4 and 4.6 ppm represent the -CH_2_-COO- protons substituted on NH_2_ of CM-Ch C_2_ and OH of CM-Ch C_6_, respectively. Similarly, the observed peak at 2.3 is for the N-C-H amine groups of chitosan that are substituted by -CH_2_COOH. A small intensity peak at 1 ppm corresponds to N-H amine groups for unsubstituted amine groups in CM-Ch. The molecular weights of Ca-CM-Ch, Sa-CM-Ch, and sy-CM-Ch were 545.2, 575.1, and 561.2 Da, respectively. These were estimated using the most significant peak from MALDI-TOF MS analysis (Figures 2(d)–2(f)).

### 3.2. CM-Ch Inhibits HepG2 Proliferation

**Figure 3:**
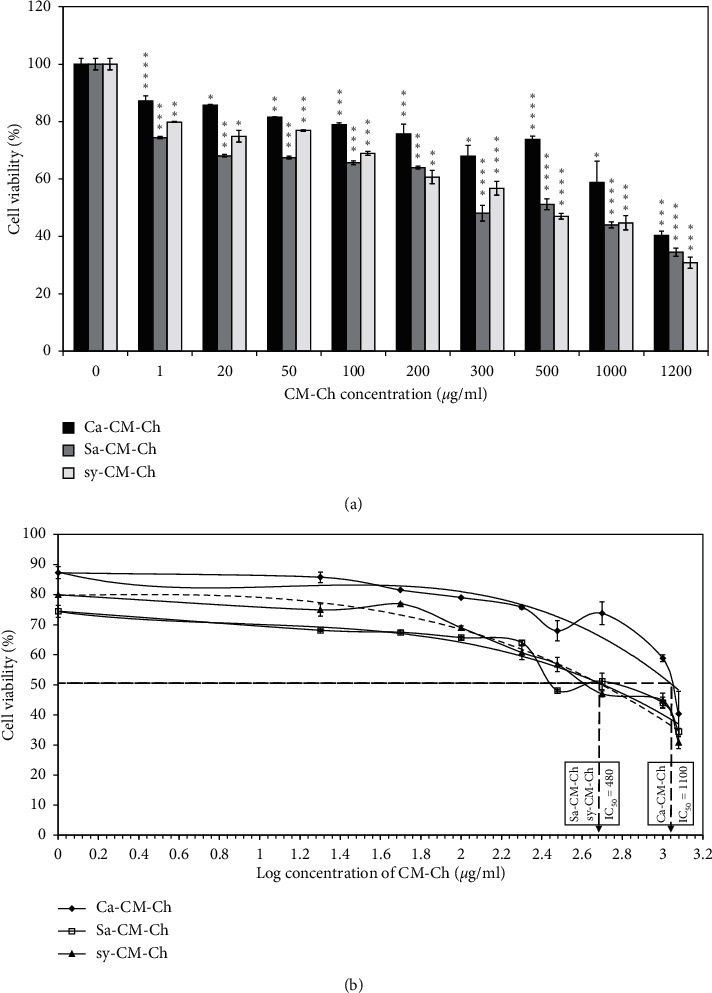
Effects of carboxymethyl chitosan (CM-Ch) on the viability of HepG2 cells. Cells were treated with CM-Ch (1–1200 *µ*g/mL) for 48 h. Controls were treated with DMSO only. (a) CM-Ch reduced HepG2 cell viability. (b) Semilogarithmic plotting of HepG2 cell viability at increasing CM-Ch concentrations was used to calculate IC_50_. Ca-CM-Ch, *Chrysomya albiceps* CM-Ch; Sa-CM-Ch, *Sarcophaga aegyptiaca* CM-Ch; sy-CM-Ch, CM-Ch of commercial chitosan. Data (*n* = 3) were presented as means ± SEM. *p* value was calculated vs. control cells: ^*∗*^*p* < 0.05, ^∗∗^*p* < 0.01, ^∗∗∗^*p* < 0.005, and ^∗∗∗∗^*p* < 0.001.

To determine the potential cytotoxic effect of the three different preparations of CM-Ch on the HepG2 cells, cell viability was evaluated using an MTT colorimetric assay. Treatment of HepG2 cells with increasing concentrations (0–1200 *µ*g/mL) of different CM-Ch compounds induced significant dose-dependent growth inhibition and reduced HepG2 cell viability after 48 h (Figure 3(a)). Semilogarithmic plotting of HepG2 cell viability percentage at increasing CM-Ch concentrations was used to calculate IC_50_. HepG2 cells showed higher sensitivity towards both Sa-CM-Ch and sy-CM-Ch than they did towards Ca-CM-Ch. Calculated IC_50_ for the three different CM-Ch extracts was 480 *µ*g/mL for either Sa-CM-Ch or sy-CM-Ch and 1100 *µ*g/mL for Ca-CM-Ch (Figure 1(b)).

### 3.3. CM-Ch Induces Morphological Changes in HepG2 Cells

**Figure 4:**
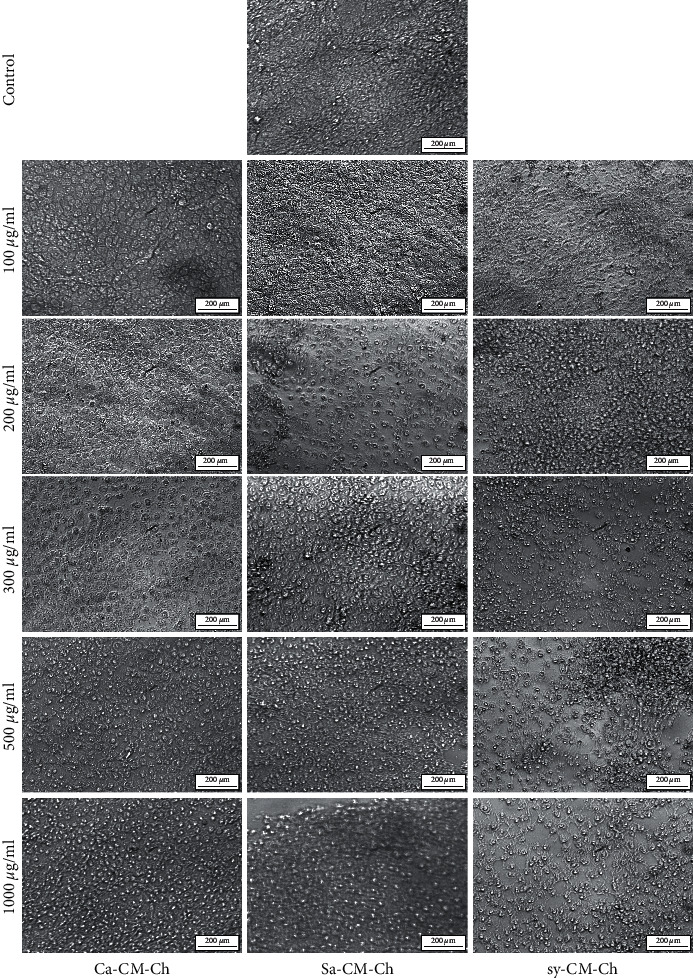
Carboxymethyl chitosan (CM-Ch) induced morphological changes in HepG2 cells. Cells were treated with the indicated concentrations of CM-Ch for 48 or 72 h (sy-CM-Ch). Representative images were shown from three independent experiments. Ca-CM-Ch, *Chrysomya albiceps* CM-Ch; Sa-CM-Ch, *Sarcophaga aegyptiaca* CM-Ch; sy-CM-Ch, commercial chitosan CM-Ch. 400×.

Treatment of HepG2 cells with increasing concentrations of CM-Ch (0–1000 *µ*g/mL) caused morphological alterations after 48 h (Figure 4). Untreated cells showed high confluency rate and appeared as normal monolayer cells. Morphological alterations observed in cells treated with CM-Ch include reduced cell dimensions, cell shrinkage, and chromatin condensation. These morphological changes become more obvious at higher doses. HepG2 cells treated with increasing concentrations of Ca-CM-Ch or Sa-CM-Ch, for 48 h, displayed characteristic apoptotic features including reduced cell dimensions and cell shrinkage. The majority of cells exhibited a rounded shape after treatment with 300 *µ*g/mL CM-Ch. Chromatin condensation was detected, and the cellular morphology of HepG2 cells was severely distorted. Treatment of HepG2 cells with higher Ca-CM-Ch or Sa-CM-Ch concentrations resulted in more distinct, clear morphological alterations with detached cells and weak cell-cell contact. HepG2 morphological changes were also observed after 72 h of treatment with sy-CM-Ch (Figure 4).

### 3.4. Apoptosis Is Induced in CM-Ch-Treated HepG2 Cells

**Figure 5:**
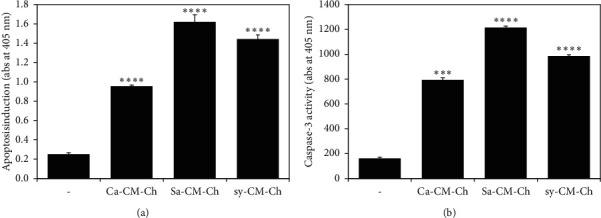
Carboxymethyl chitosan (CM-Ch) induced caspase-dependent apoptosis in HepG2 cells. Cells were either untreated or treated with the indicated CM-Ch (300 *µ*g/mL) for 48 h. Lysed cells were subjected to (a) enzyme-linked immunosorbent apoptosis assay to measure histone release as an indication of apoptosis; (b) caspase-3 activity assay. Each assay was performed in triplicate, and the standard error of the mean (SEM) was calculated. Data were presented as mean ± SEM. *p* value was calculated vs. control cells: ^∗∗∗^*p* < 0.005 and ^∗∗∗∗^*p* < 0.001. Ca-CM-Ch, *Chrysomya albiceps* CM-Ch; Sa-CM-Ch, *Sarcophaga aegyptiaca* CM-Ch; sy-CM-Ch, CM-Ch of commercial chitosan.

We quantified histone release to assess apoptosis and to confirm inhibition of cell viability in HepG2 cells treated with Ca-CM-Ch, Sa-CM-Ch, and sy-CM-C. Apoptosis induction was significantly higher in cells treated with CM-Ch than in untreated cells (Figure 5(a)). The highest level of apoptosis was detected in Sa-CM-Ch-treated HepG2 cells.

### 3.5. CM-Ch Compounds Induce Caspase-3 Activity in HepG2 Cells

Caspase activation characterizes the final pathway of apoptotic signal transduction. Detection of cleaved caspase-3 (activated caspase-3) confirms apoptosis induction. The three different CM-Ch compounds significantly increased the activation of caspase-3 in HepG2 cells (Figure 5(b)). Sa-CM-Ch treatment resulted in the highest caspase-3 activity of all tested CM-Ch compounds.

### 3.6. CM-Ch Treatment Shows No Signs of Toxicity and No Changes in Biochemical Markers of Albino Rats

We used the Registry of Cytotoxicity (RC) method to generate a model to forecast the lethal dose (LD_50_) of each CM-Ch treatment. LD_50_ values were calculated from IC_50_ values, as reported previously [26], using the RC regression equation:

(5) $$\begin{array}{c} \text{log}\left(LD_{50}\right)=0.435*\text{log}\left(IC_{50}\right)+0.625. \end{array}$$

**Figure 6:**
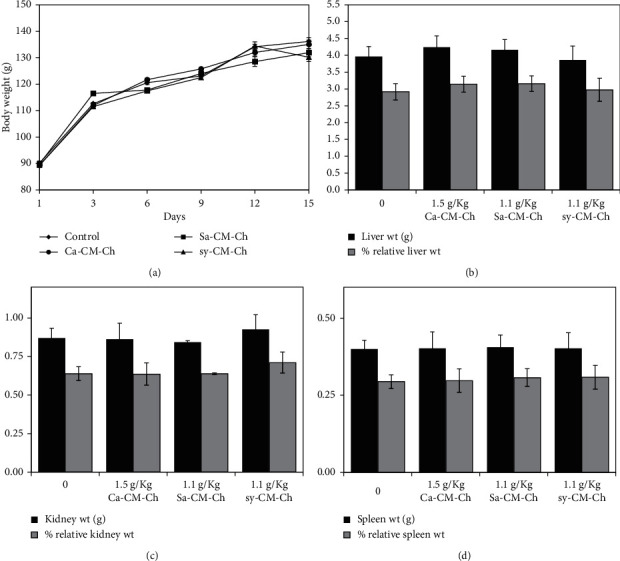
CM-Ch treatment did not affect body weight, organ weight, and percentage of organ weight relative to body weight in rats. Animals were treated with a single oral dose of either Ca-CM-Ch (1.5 g/kg), Sa-CM-Ch (1.1 g/kg), sy-CM-Ch (1.1 g/kg), or saline (1 mL/kg) as a control. Body weight, organ weight, and percentage of organ weight relative to body weight were measured after 15 days of treatment. Data were represented as mean ± SEM of five animals. Ca-CM-Ch, *Chrysomya albiceps* CM-Ch; Sa-CM-Ch, *Sarcophaga aegyptiaca* CM-Ch; sy-CM-Ch, CM-Ch of commercial chitosan. Body and organ weights and percentage of kidney, liver, and spleen organ weight relative to body weight did not significantly differ between treated groups and controls.

Calculated LD_50_ values were 3.1 g/kg for Ca-CM-Ch and 2.2 g/kg for Sa-CM-Ch and sy-CM-Ch. To evaluate the toxicity of CM-Ch, half of LD_50_ for each CM-Ch was orally administered to albino rats at the start of the 15-day experimental period. None of the treated rats died, and there were no signs of toxicity or behavioral changes when compared to the untreated control group. Furthermore, no conspicuous pathological changes were detected at necropsy in any of the rats at the end of the experimental period. Changes in mean body weights of the treated groups did not significantly differ from those in the untreated control group (Figure 6). Body and organ weight and comparisons revealed no significant differences between treatment groups and controls (Figure 6).

**Figure 7:**
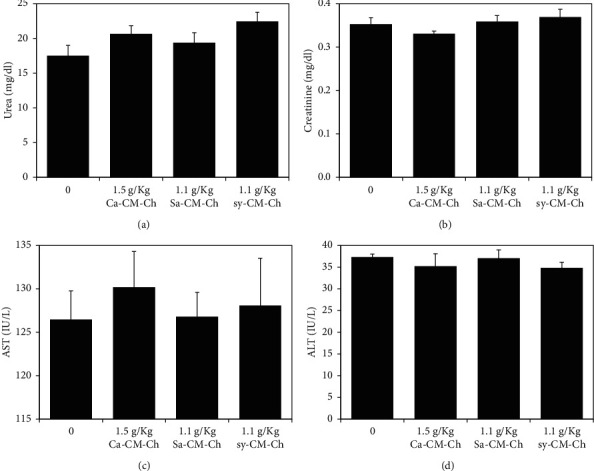
Kidney and liver biochemical parameters in rats treated with CM-Ch. Animals were treated with a single oral dose of either Ca-CM-Ch (1.5 g/kg), Sa-CM-Ch (1.1 g/kg), sy-CM-Ch (1.1 g/kg), or saline (1 mL/kg) as a control. Biochemical parameters of the kidney (serum urea and creatinine) and liver (AST and ALT) were measured after 15 days of treatment. Data were presented as mean ± SEM of five animals. Ca-CM-Ch, *Chrysomya albiceps* CM-Ch; Sa-CM-Ch, *Sarcophaga aegyptiaca* CM-Ch; sy-CM-Ch, CM-Ch of commercial chitosan. No significant difference in liver and kidney parameters was observed in treated groups and controls.

Kidney and liver functions were also investigated to further evaluate the biological safety of the three different CM-Ch compounds. Kidney function was evaluated by measuring urea and creatinine levels in treated and untreated groups. No significant difference in kidney parameters was observed between treated and untreated control groups (Figure 7). Serum AST and ALT activities were measured to assess liver function. No significant difference in liver parameters was observed in treated groups and untreated groups (Figure 7).

## 4. Discussion

The omnipresence of insects in any type of ecological system, from waterways to exceedingly infected environments, enables scientists to explore Arthropoda for different therapeutic agents [30]. Insects and their derived compounds may also have bioactive functions and should be considered a viable source of therapeutically effective medicines [31, 32]. The use of natural products has been the foremost successful approach to enhancing antitumor activity by modulating basic mechanisms [33]. This study was designed to explore the bioactivity of insect chitosan as a therapeutic agent against liver cancer. A low molecular weight, water-soluble chitosan derivative, CM-Ch, was prepared to assess its effect on the proliferation of HepG2 HCC cells and to evaluate its toxicity in rats.

The extraction of chitosan involved several steps, including field collection and laboratory rearing of the insect species, *C. albiceps* and *S. aegyptiaca*, followed by chitosan processing, including deproteination and deacetylation. The cuticle of crustaceans is typically composed of chitin in a mineral and protein matrix [6, 34]. In contrast, the cuticle of insects consists of chitin in a matrix with lipids, cuticular proteins, and additional compounds [35]. Most insects contain insignificant amounts of minerals in their cuticle, so the demineralization process was not required [36, 37]. The average molecular weights of chitosan extracted from *C. albiceps* and *S. aegyptiaca* were lower than those of commercial chitosan. Moreover, chitosan extracted from *C. albiceps* and *S. aegyptiaca* had a relatively higher DD than did commercial chitosan, which is promising for further applications given that the DD of chitosan is proportional to its biological activity [38, 39].

The characteristic properties of chitosan extracted from *C. albiceps* and *S. aegyptiaca* were identified using FTIR analysis. The spectra resulting from *C. albiceps* and *S. aegyptiaca* larval chitosan were similar to those of chitosan extracted from *Chrysomya megacephala* larvae, *Musca domestica* larvae, chrysalides of the silkworm, and the exoskeletons of crabs and shrimps. The positions and intensities of characteristic peaks were almost identical to those reported previously [21, 40, 41]. The solubility of chitosan in moderate acidic conditions confers a severe disadvantage to many of its biologically possible applications [42]. Therefore, carboxymethylation was used to enhance chitosan processability and to strengthen some of its biological characteristics [43, 44]. This step improved the biological activity of chitosan and may potentiate its activity as an antitumor agent in the future. The peaks observed at 1614 and 1594 cm^−1^ may be attributed to axial stretching of the C=O bond of the acetamide (-CONH-) group of acetylated chitosan units [45]. The peak, characteristic for amine, at 3400 cm^−1^ was less intense in *C. albiceps* than in *S. aegyptiaca* which was consistent with the DD% results [46]. Additionally, the appearance of new peaks at 1728 and 1638 cm^−1^ in the spectra of CM-Ch prepared from chitosan extracted from both flies may be attributed to symmetric and antisymmetric stretching of the carboxylate group C=O in CM-Ch as reported previously [45]. The absence of the 1728 cm^−1^ peak in the spectrum of the CM-Ch prepared from commercial chitosan may be due to the nature of the CM-Ch produced, pure O-CM-Ch, while both O-CM-Ch and N-CM-Ch are formed in CM-Ch preparations from insect chitosan, potentially causing an N-H deformation leading a peak shift to the 1728 range [46]. This difference may have arisen because of the temperature used during the carboxylation process in this work. CM-Ch structural complexity is difficult to characterize and requires clear identification [47]. Consequently, the infrared spectra of chitosan and CM-Ch were compared using ^1^H NMR spectroscopy to reveal the hydrophilic characteristics of CM-Ch compared to parental chitosan.

Despite the joint efforts of governments and researchers globally, the incidence of HCC has continued to rise over the last two decades [48]. Previous studies of chitosan and chitosan derivatives indicate that the inhibition of proliferation in tumors *in vivo* following chitosan treatment occurs by immune system stimulation through increasing lymphokine production and enhancing lymphocyte activity [49]. Here, we used HepG2 cells as a model for HCC to investigate the potential cytotoxic effects of CM-Ch. Treatment of HepG2 cells with CM-Ch inhibited cell growth. To investigate the possible cytotoxic and antiproliferative effects of the three different CM-Ch extracts, Ca-CM-Ch, Sa-CM-Ch, and sy-CM-Ch on HepG2 cells, we performed MTT assays. Our results show that cell viability was reduced in a dose-dependent manner after 48 hrs of treatment. Similarly, another family of chitin and chitosan derivatives was shown to have antiproliferative effects on SMMC-7721 HHC cells [50].

The proliferation of A549 (human lung adenocarcinoma) and WiDr (colon adenocarcinoma) cancer cells was significantly inhibited by low-molecular weight chitosan extracted from mayfly, commercial low-molecular weight chitosan (LMWCc), and commercial medium-molecular weight chitosan (MMWCc) [51]. Additionally, LMWCc exhibited higher antitumor activity towards HeLa (human epithelial cervical cancer) cells than did mayfly chitosan or MMWCS. Hence, the molecular weight, DD, and the animal source from which chitosan is obtained influence the activity of chitosan against cancer cell lines [51]. Here, low-molecular weight CM-Ch from different sources exhibited strong antiproliferation activity towards the HepG2 cell line with different IC_50_. While few studies have evaluated the inhibitory effect of CM-Ch on tumor cell lines, a previous study reported that CM-Ch has a slight inhibitory effect on proliferation in SGC-7901 (human gastric cancer), Bel-7402 (human hepatocellular carcinoma), and HeLa cancer cell lines only at high concentrations, up to 5 mg/mL [52]. Similar results showed that concentrations of 1 and 2 mg/mL CM-Ch did not reduce proliferation in four cancer cell lines, OSRC-2 (renal cell carcinoma), SGC-7901, NCI-H1650 (lung adenocarcinoma), and HT-29 (colon adenocarcinoma), although signiﬁcant reduction in cell viability was observed at higher concentrations [53]. The CM-Ch used in this study produced significant antiproliferative effects within a low concentration range and could be considered a promising anticancer reagent. Indeed, the carboxymethylation process could modify the biological characteristics of chitosan, and CM-Ch may show remarkably different and/or enhanced biological activities than does original chitosan.

The induction of apoptosis is an important pathophysiological strategy through which an ideal antitumor agent acts to stop cancer cell growth [54]. Apoptosis is typically characterized by morphological alterations and biochemical hallmarks in cells [48]. Morphological observation of CM-Ch-treated HepG2 cells revealed rounded-up cells with typical apoptotic phenotypes including cell shrinkage and chromatin condensation that may be due to the growth arrest and apoptotic induction after CM-Ch treatment. The apoptosis-related morphological changes of HepG2 cells treated with Ca-CM-Ch and Sa-CM-Ch were clearly observed after 48 h, while those treated with sy-CM-Ch exhibited similar morphological alterations after 72 h. Additionally, HepG2 cells were more sensitive towards CM-Ch extracted from insects than CM-Ch derived from commercial chitosan. The observed differences in the activity of different CM-Ch preparations towards HepG2 cells could be because they were extracted from different animal sources. *C. albiceps* is a viviparous insect, while *S. aegyptiaca* is a larviparous insect. Differences in biology of oviposition and fecundity of females could affect the composition of chitin in the offspring, which could then affect the biological activities. However, this requires further investigation.

Nucleosomal histones are released from apoptotic chromatin which, kinetically, matches well with DNA fragmentation [55]. Our results indicate that the three different CM-Ch compounds tested significantly induce the release of histone from HepG2 cells. Apoptosis is regulated via a sequence of signal cascades, the intrinsic pathway, and the extrinsic pathway [56]. Both pathways are key molecular signaling pathways, involved in apoptosis and triggering the enzymatic caspase-cascade signaling system, leading to several proteolytic events mediating programmed cell death [57]. Caspase-3 is common to both intrinsic and extrinsic apoptosis pathways [56]. The manifestation of the hitting effect of caspase-3 on HepG2 cells, herein, was to confirm the induction of apoptosis by CM-Ch treatment, demonstrating a significant elevation of the caspase-3 activity after CM-Ch treatments. These results indicate that the observed inhibition of HepG2 cell growth following CM-Ch treatment may be attributed to the induction of apoptosis via caspase-3 activation. The highest caspase-3 activity was observed in cells treated with Sa-CM-Ch, and this bioactivity may be ascribed to its high DD [38]. Consistently, chitosan induced apoptosis in bladder tumor cells through caspase-3 activation [58]. Further studies are required to focus on the signaling pathway through which CM-Ch triggers apoptosis in HepG2 cells.

Several studies have revealed the biological safety of CM-Ch *in vitro*. Treatment of human umbilical vein endothelial cells (HUVECs) with different concentrations of CM-Ch showed no significant decrease in cell viability after 24 and 48 hrs of incubation and that 0.5–1.5 mg/mL of CM-Ch was nontoxic to HUVECs [59]. Similarly, CM-Ch was nontoxic to L02 human normal liver cells [52]. Furthermore, a previous study on chitin derivatives showed that *O*-carboxymethyl chitin exhibited no cytotoxic activity on MRC-5 human normal lung fibroblastic cells at concentrations lower than or equal to 2 mg/mL [60]. Determining toxicity is a fundamental step in the evaluation of the safely of CM-Ch compounds. Therefore, we assessed the safety of insect-derived CM-Ch by studying the biochemical parameters in rats. Previous studies reported that CM-Ch had insignificant toxicity in rats [27, 61]. We assessed the toxic effects of treatment with CM-Ch by monitoring noticeable morphological and behavioral signs and weight changes and by determining the activity of some key liver and kidney enzymes. The lack of changes in AST, ALT, urea, and creatinine levels for all investigated CM-Ch compounds shows that there is no evidence of any liver or renal toxicity in the treated animals. Presumptively, it may be concluded that the oral administration of CM-Ch, prepared from chitosan that was extracted from the larvae of *C. albiceps*, *S. aegyptiaca*, and commercial crustacean chitosan, to rats is safe and does not affect any of the investigated biochemical parameters.

## 5. Conclusion

In drug discovery from natural sources, insects are one of the least investigated groups. Insect therapeutics represents a significant untapped field of novel pharmaceutics. CM-Ch from insect sources could be a potent and inexhaustible commercially produced agent. Indeed, CM-Ch is effective to reduce cell viability and growth in HepG2 cells by inducing apoptosis without exerting any toxic effects *in vivo*. More investigations will validate the compelling chemopreventive capacities of CM-Ch in other cancer models. If chitosan or its derivatives demonstrate chemopreventive capabilities *in vivo*, they might be useful therapeutic agents for liver malignancy.

## Figures and Tables

**Table 1:** FTIR vibration bands of chitosan extracted from *Chrysomya albiceps* and *Sarcophaga aegyptiaca* and FTIR vibration bands of carboxymethyl chitosan prepared from chitosan extracted from *C. albiceps* (Ca-CM-Ch), *S. aegyptiaca* (Sa-CM-Ch), and commercial chitosan (sy-CM-Ch).

Functional group		Frequency (cm^−1^)	
		*C. albiceps* chitosan	*S. aegyptiaca* chitosan
Stretching vibration of N-H and -OH		3425	3435
Asymmetric C-H stretching vibration		2926	2928
Symmetric C-H stretching vibration		2767	2766
Stretching vibration of C=O bond of acetamide		1564	1614
Vibration of the (C-N) amine group		1446	1411
Stretching vibration of C-O		1080	1076
Characteristic group	Ca-CM-Ch	Sa-CM-Ch	sy-CM-Ch

Vibration of O-H of carboxylic groups	3392	3456	3423
Deforming bending vibration of N-H	1732	1638	1728
Vibration of C=O of carboxylic groups	1417	1367	1404
Vibration of the C-O-C bond	1082	1087	1084

## Data Availability

The data used to support the findings of this study are available from the corresponding author upon request.
